# SAW Torque Sensor Gyroscopic Effect Compensation by Least Squares Support Vector Machine Algorithm Based on Chaos Estimation of Distributed Algorithm

**DOI:** 10.3390/s19122768

**Published:** 2019-06-20

**Authors:** Wei Han, Xiongzhu Bu, Yihan Cao, Miaomiao Xu

**Affiliations:** Nanjing University of Science and Technology, School of Mechanical Engineering, 200 Xiaolingwei, 210094 Nanjing, China; hanwei1114@njust.edu.cn (W.H.); caoyihan1218@njust.edu.cn (Y.C.); ck_xumiao@njust.edu.cn (M.X.)

**Keywords:** SAW, torque sensor, gyroscopic effect compensation, CEDA-LSSVM

## Abstract

As this study examined the issue of surface acoustic wave (SAW) torque sensor which interfered in high rotational speed, the gyroscopic effect generated by rotation was analyzed. Firstly, the SAW coupled equations which contained torque and rotation loads were deduced, and the torque calculation error caused by rotation was solved. Following this, the hardware of the SAW gyroscopic effect testing platform and the turntable experiment were designed to verify the correctness of the theoretical calculation. Finally, according to the experimental data, the gyroscopic effect was compensated by multivariate polynomial fitting (MPF), Gaussian processes regression (GPR), and least squares support vector machine algorithms (LSSVM). The comparison results showed that the LSSVM has the obvious advantage. For improving the function of LSSVM model, chaos estimation of distributed algorithm (CEDA) was proposed to optimize the super parameters of the LSSVM, and numerical simulation results showed that: (1) CEDA is superior to traditional estimation of distributed algorithms in convergence speed and anti-premature ability; (2) the performance of CEDA-LSSVM is better than genetic algorithms (GA)-LSSVM and particle swarm optimization (PSO)-LSSVM. After compensating by CEDA-LSSVM, the magnitude of the torque calculation relative error was 10^−4^ in any direction. This method has a significant effect on reducing gyroscopic interference, and it lays a foundation for the engineering application of SAW torque sensor.

## 1. Introduction

In mechanical transmission systems, torque is one of the most valuable mechanical quantities to evaluate the system performance, and to reflect both power and condition of the machinery. Torque measurements with high accuracy have the essential prerequisites for operation, monitoring, and fault diagnosis [1,2]. However, it is hard to measure under the condition with rotation, narrow spaces, and high vibrations by traditional methods [3]. The surface acoustic wave (SAW) torque sensor is the emerging technology, and it has the benefits of passive, wireless, high sensitivity, small size, high stability, and near digital signal output which facilitates the subsequent signal processing. The mature development of that technology will be destined to replace the traditional torque sensor [4]. 

The accuracy of the SAW torque sensor is affected by the environment. One of the factors is the gyroscopic effect caused by rotation [5]. At present, the research on gyroscopic effect interference and the compensation method of the SAW torque sensor is still in the exploratory stage, and the corresponding research report cannot be found. Xiaojun Ji et al. [6] performed research on the novel demodulation system. In this system, the equipment has the fuller dynamic response range and more effective length of the signal which utilized the clipping amplifier, and a correlation extension algorithm was developed to suppress the noise and to improve the resolution of frequency estimation. V. Kalinin et al. [7] established a large diameter radio frequency rotary coupler and wireless rotational angle SAW measurement system. The new clamp-on the SAW sensing element did not need to transform the shaft. However, the influence of gyroscopic effect on the SAW sensors was not considered.

**Figure 1:**
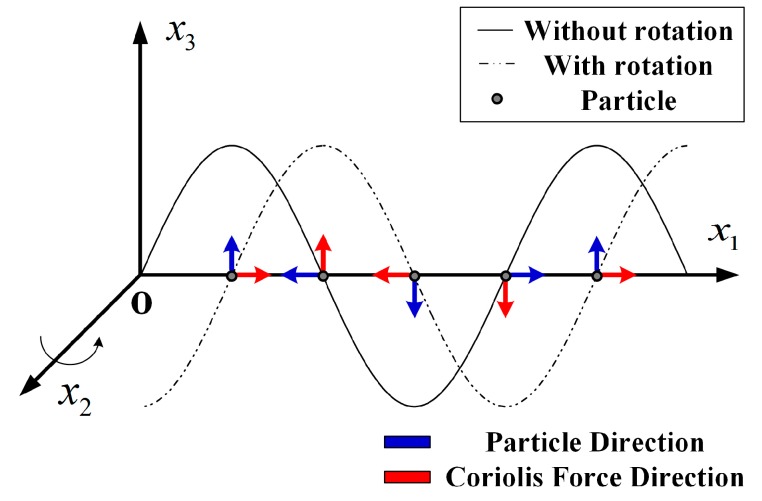
The effect of gyroscopic on the surface acoustic wave (SAW) propagation.

The gyroscopic effect is a phenomenon that the propagation parameters of the SAW would be changed when the SAW device rotates [8]. Taking the particles on the SAW device surface as the observed objects, the gyroscopic effect on the SAW is shown in Figure 1. As the SAW propagated along the *x*_1_ axis, the medium rotated uniformly around the *x*_2_ axis, and only the Coriolis inertial force on the particle is shown. The original particle motions are similar to the simple harmonic oscillation perpendicular to the surface. When the rotation has happened, the normal motion is affected, and a new running wave was shifted a quarter of the wavelength and coupled with the original wave, which affected the propagation characteristics of the SAW. It was inconspicuous under static or low rotational situations, which cannot be ignored with high speed [9]. It is of great significance to work on the compensation method of the SAW torque sensor gyroscopic effect for improving the accuracy and broadening the scope of application.

Generally speaking, the compensation methods for sensor interference are divided into the hardware compensation method and the software compensation algorithm. At present for SAW devices, the research on compensation of environmental factors has focused mostly on the hardware compensation method. V. Kalinin et al. [10] developed a wireless temperature and torque sensor based on three one-port SAW resonators. The three SAW resonators were fabricated on the same substrate in a certain array mode to realize the interference compensation and the ambient temperature calculation of the device at the same time. Wen Wang et al. [11] undertook research on the SAW vibration sensor with temperature compensation. Two sets of SAW sensors were fabricated on Y-cut quartz cantilever beam, one of which was arranged at the far end of proof mass on the beam to obtain maximum sensitivity, and another group completed the temperature compensation with differential structure. It can be seen that the hardware compensation method not only increases the hardware expenditure at the front-end of the sensor, but also troubles the detection link in the reader. Meanwhile, the compensation structure cannot be exactly the same in the actual processing. The sensor calibration is difficult, and the accuracy and compensation effect are easily affected by the processing error. The gyroscopic effect caused by rotation is a spatial problem, which cannot guarantee that the compensation structure can be in the same rotating axis as the sensitive structure. Therefore, the hardware method is not suitable for the gyroscopic effect compensation of the SAW sensors, and appropriate software compensation algorithms should be designed to compensate for the interference error.

High sensitivity is the remarkable advantage of the SAW sensor, however, sensing too many physical quantities also restricts the development of the SAW sensor. In the normal torque measurement environment, the varying disturbance of temperature and rotation is ubiquitous. Hence, the strengths of the SAW sensor may be truly highlighted only by compensating for the disturbance. It is significant for all non-speed sensitive SAW devices. In this paper, the influence of the SAW torque sensor with gyroscopic effects under high rotating situations is studied. The transfer model of the SAW torque sensor in rotational process is deduced, and the verification experiment of the gyroscopic effect is designed. Based on the experimental data, the gyroscopic effect was compensated by the least squares support vector machine algorithms (LSSVM) to improve the accuracy of the torque measurement. In order to improve the performance of the LSSVM, the chaos estimation of distributed algorithm (CEDA) is proposed to optimize the regularization parameter and kernel parameter. The numerical simulation results show that the LSSVM algorithm based on the CEDA parameter optimization can compensate the interference of the gyroscopic effect on the SAW torque sensor.

## 2. Model of SAW Torque Sensor under High Rotational Speed

**Figure 2:**
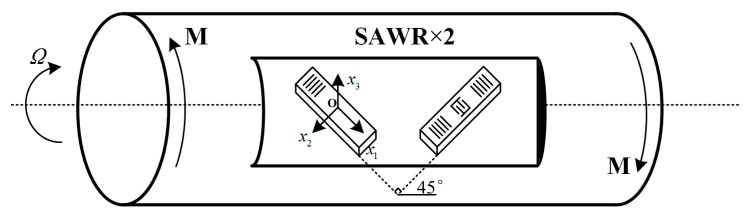
The SAW torque elements installation diagram.

The installation of SAW torque elements is shown in Figure 2, where M is the applied torque. Based on the round shaft torsion plane hypothesis in mechanics of materials and corresponding calculations, the torque elements are arranged on the surface of the working axis and at an angle of ±45° to the shaft axis. As shown in Figure 2, SAW propagates along the *x*_1_ axis on the plane of *x*_3_ = 0 in *Ox*_1_*x*_2_*x*_3_ coordinate, and the working shaft revolves around the axis.

When torque and rotation are applied to the shaft, the angular velocity of the medium relative to the inertial space is ***Ω***, the particles at the surface are subjected the stress ***σ*** by torque; the Coriolis force −2*m**Ω***× ***v***; and the centrifugal force −*m**Ω***× (***Ω*** × ***R***); the *v* and the ***R*** are the instantaneous velocity vector and instantaneous position vector of a particle on the SAW device surface relative to the rotating axis inertial coordinate system. Scalar forms can be applied by using generalized Hooke’s law and the vector algorithm. The SAW propagation characteristic on the dynamic deformable substrate with torque and rotation load is defined as Equation (1) [12,13].

(1) $$\left\{ \begin{array}{l} \rho \frac{\partial^{2}u_{i}}{\partial t^{2}}=(C_{ijkl}+{\hat{C}}_{ijkl})\frac{\partial^{2}u_{k}}{\partial x_{l}\partial x_{j}}+e_{kij}\frac{\partial^{2}\phi }{\partial x_{k}\partial x_{j}}-2\rho \zeta_{ijk}\Omega_{j}\frac{\partial u_{l}}{\partial t}-\rho (\Omega_{i}\Omega_{j}u_{j}-\Omega_{j}^{2}u_{j})\\ e_{jkl}\frac{\partial^{2}u_{k}}{\partial x_{l}\partial x_{j}}-\varepsilon_{jk}\frac{\partial^{2}\phi }{\partial x_{k}\partial x_{j}}=0 \end{array}\right.$$

In Equation (1), *ρ* is the density, *C_ijkl_*, *e_jkl_*, and *ε_jk_* are the stiffness, the piezoelectric modules, and the components of permittivity of the material, *ζ_ijk_* is the permutation, *Ω_j_* is the angular velocity of the rotating devices, *u* and *ϕ* are the displacement and the electrical potential. Einstein’s summation rule is used, and *i*, *j*, *k*, *l* are changed from 1 to 3. ${\hat{C}}_{ijkl}$ is the perturbation bias, which depends on the torque is defined as Equation (2).

(2) $${\hat{C}}_{ijkl}=T_{ik}\delta_{jl}+C_{ijklmn}E_{mn}+C_{ijnk}\frac{\partial W_{l}}{\partial x_{n}}+C_{inkl}\frac{\partial W_{j}}{\partial x_{n}}$$

where *m*, *n* = 1, 2, 3, *T_ik_* is the stress tensor, *δ_jl_* is the Kronecker delta, *C_ijklmn_* and *C_ijnk_* are the third-order and the second-order elastic constants, *E_mn_* is the biasing strain, ∂*W_l_*/∂*x_n_* is the displacement gradient component.

The solutions of the SAW motion equations have the form of Equation (3).

(3) $$\left\{ \begin{array}{l} u_{i}=A_{k}\exp[j(\omega t-\beta l_{p}x_{p})]\\ \phi =\phi_{0}\exp[j(\omega t-\beta l_{p}x_{p})] \end{array}\right.$$

where *A_k_* and *ϕ_0_* are the amplitudes, *β* = *ω*/*v* is the wave number, *v* is the phase velocity of SAW, *l_p_* is the direction cosine and *p* = 1, 2, 3. Substituting Equation (3) into Equation (1), according to the direction and the plane of the SAW propagation, *l_p_* can be expressed as *l*_1_ = 1, *l*_2_ = 0, *l*_3_ = *η*, *η* is the attenuation coefficient of the SAW, the Christoffel equations with torque and rotation are derived as Equation (4).

(4) $$\left\{ \begin{array}{l} A_{k}l_{l}l_{j}({\hat{C}}_{ijkl}+C_{ijkl})-A_{k}\rho v^{2}\delta_{ik}-2A_{k}\rho v^{2}j\zeta_{ijk}\Omega_{j}/\omega +e_{jkl}l_{k}l_{j}\phi_{0}=0\\ \phi_{0}=A_{k}\frac{e_{jkl}l_{l}l_{j}}{\varepsilon_{jk}l_{k}l_{j}} \end{array}\right.$$

The ratio of the centrifugal force and the Coriolis force is proportional to *Ω/ω*, therefore, the contribution of the centrifugal force is far less than the Coriolis force to the SAW devices because of *Ω/ω*≪1, and the centrifugal force *A_k_δ_ik_ρv^2^*(1+*Ω_j_*^2^+*Ω_i_Ω_j_*)/*ω*^2^ is ignored in Equation (4). The 2*A_k_ρv*^2^*jζ_ijk_Ω_j_*/*ω* term represents the influence of the Coriolis force on the SAW propagation, which has been neglected by previous SAW torque sensors.

**Figure 3:**
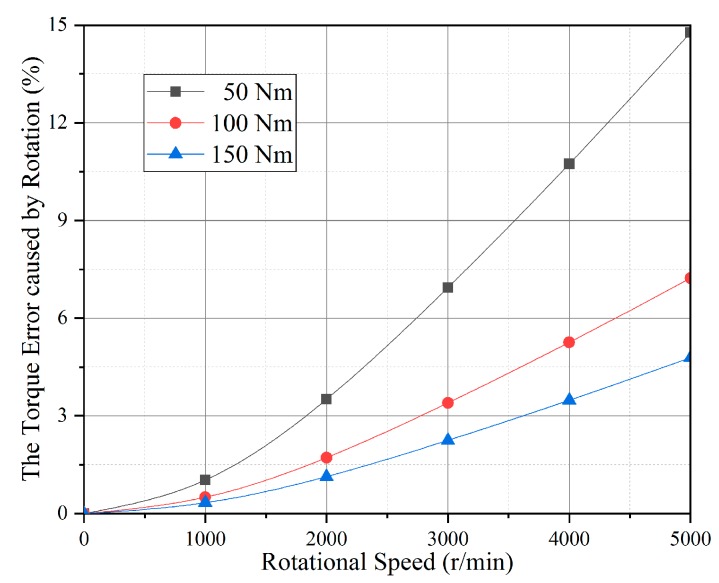
The torque calculation error caused by rotation.

If the **Ω** direction coincides with the SAW propagating direction, and taking a new piezoelectric material Lanthanum Gallium Silicate (LGS) as an example, Equation (4) is solved by the determinant absolute value method of the boundary conditions coefficient [14]. The Euler angle of the LGS is (0°, 140°, 24°) which has better temperature performance, and other parameters are considered as this cut type. Firstly, the resonance frequency shifts under 50 Nm, 100 Nm and 150 Nm are calculated. Then, the resonance frequency shifts with the gyroscopic effect under the same torque are deduced. Finally, the torque calculation error caused by the rotation is plotted as Figure 3.

As shown in Figure 3, the higher the rotation speed, the more error the rotation makes. The torque calculation error reaches 14.8% when the torque and the rotational speed are 50 Nm and 5000 r/min. Therefore, for the accuracy of the sensor, the gyroscopic effect in Equation (4) cannot be ignored, and the compensation model must be established.

## 3. Verification Experiment of Gyroscopic Effect on SAW Torque Sensor

**Figure 4:**
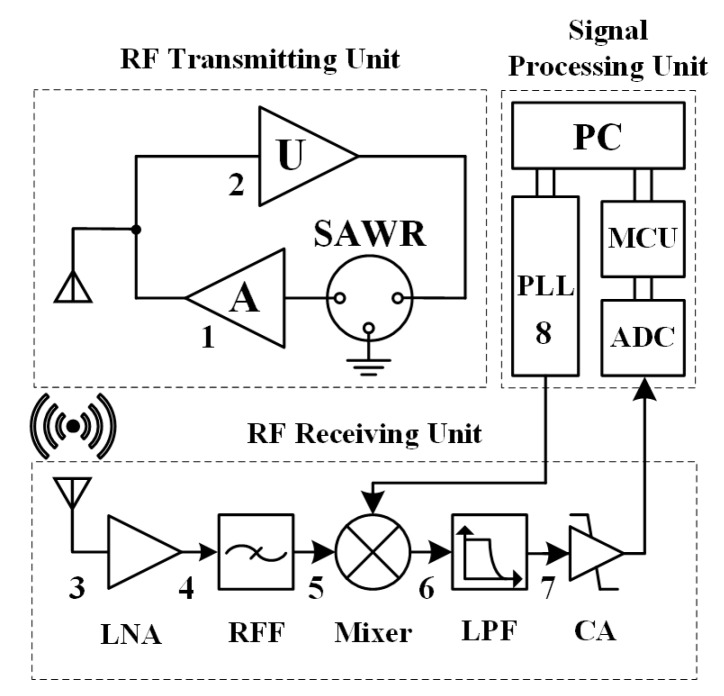
The hardware components diagram of the gyroscopic effect testing system.

Based on the analysis of the SAW sensor measurement theory, the experimental system was designed to verify the gyroscopic effect on the SAW sensor. Considering the magnitude of the gyroscopic effect, the environmental factors of the experiment, and the accuracy of the existing frequency estimation algorithm, the torque was not loaded during the experiment. The wireless passive SAW torque elements were transformed into wireless active resonators, which improved the SNR and the effective signal length for high accuracy of the gyroscopic effect test. The hardware components diagram of the testing device is shown in Figure 4.

**Figure 5:**
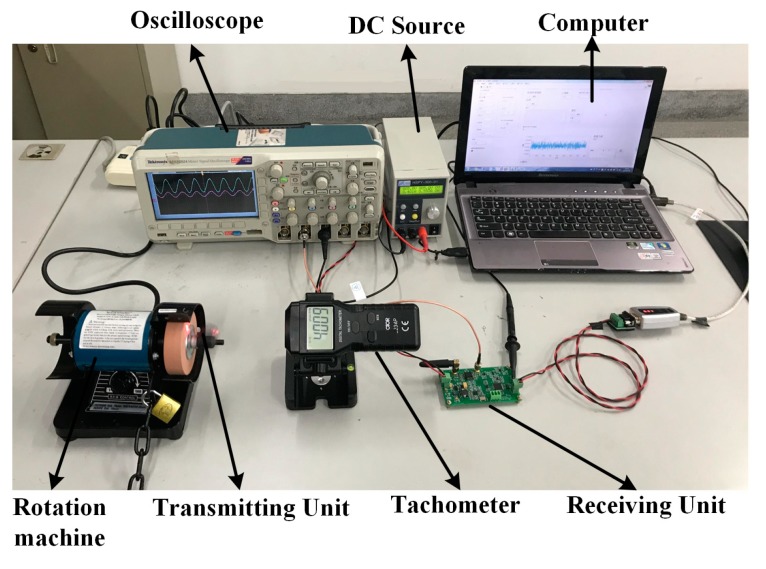
The gyroscopic effect verification platform.

A grinding machine with adjustable rotational speed from 0 to 10000 r/min was reformed and matched with a tachometer as the standard rotation generating device. The rotational speed was adjusted by the knob according to the tachometer. The RF transmitting unit was connected with the grinding machine axis through the sleeve, and the rotational axis direction can be coincided with the three axes of the SAW device coordinate system by adjusting the matching mode with the sleeve. The resonance frequency shifts caused by the gyroscopic effect was measured. The gyroscopic effect verification platform is shown in Figure 5. The substrate material of the SAW torque sensor was LGS, and the resonant frequency was 433 MHz. The circuit PCB board of receiving unit contained other hardware parts except computer.

**Figure 6:**
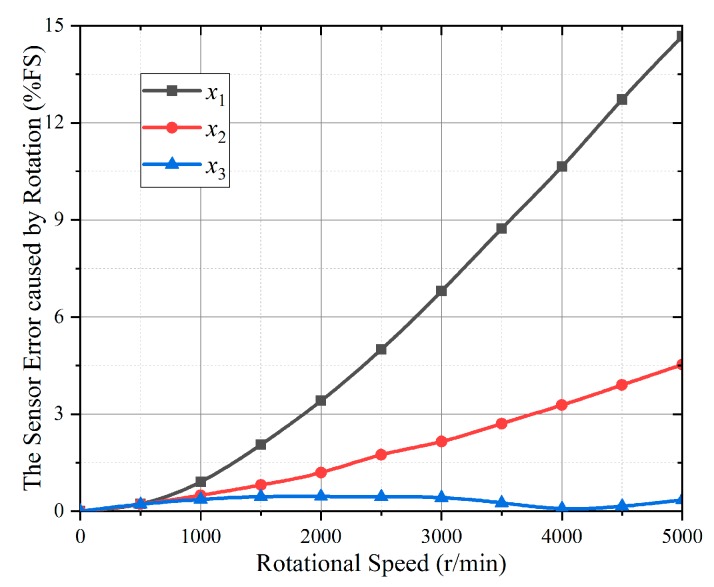
The torque calculation error caused by the rotation in *x*_1_, *x*_2_, and *x*_3_ directions.

The test rotational speed ranged from 0 to 5000 r/min with 500 r/min adjusting step. The resonance frequency shifted values of the SAW torque sensor which were measured by the test system after the rotational speed was stabilized. The torque calculation error caused by rotation and the rotational speed curve are shown in Figure 6.

As shown in Figure 6, the trend of experimental results in *x*_1_ and *x*_2_ directions are consistent with the theoretical calculation, which can verify the correctness of the theoretical calculation. The error caused by the rotation affected the accuracy of the torque calculation greatly, which must be compensated.

The data of the resonance frequency shift were not completely consistent with the theoretical values. After analysis, the reasons are as follows: (1) The RF transmitting unit and fixture are not the circular axisymmetric model that causes the rotational mass center shifting and the unstable rotational speed; (2) It is difficult to ensure that the expectant direction completely coincides with the rotating axis in the installing process; (3) The torque load is not strictly linear with the rotation load, and only applying the rotation may affect the accuracy of the model. 

## 4. The LSSVM Based on the CEDA

The complexity of the SAW transfer model with gyroscopic effect is vast, therefore, there are many problems in real-time torque calculations by substituting the resonance frequency shift and rotational speed in Equation (4), such as, large amounts of calculations and slow responses, which cannot be applied in industry. Therefore, a rotational compensation algorithm should be established to calibrate. For the torque sensor, it is expected to calibrate several points in the measurement range to complete the compensation model parameter estimation of the sensor [15]. The key to solving this issue is establishing the error compensation model with a small sample size and high accuracy.

### 4.1. The Least Square Support Vector Machine

LSSVM is an extension of the support vector machine (SVM). It improves the generalization ability through the principle of structural risk minimization, and solves practical problems such as small samples, non-linearity, high dimension, local minima, etc. It has been applied in pattern recognition, signal processing, function approximation and other fields [16].

Suppose {*x*(*i*), *y*(*i*)} (*i*=1,2,…*n*) are the data sets for training with the input *x*(*i*) ∈ *R^m^*, and the output *y*(*i*) ∈ *R*, then the SVM model can be described as Equation (5).

(5) $$y=\omega^{T}\varphi (x)+b$$

where the *φ*(·) is the nonlinear mapping. By using the squared error *ξ_i_* as the loss function, the optimization problem is as follows as Equation (6).

(6) $$\begin{array}{c} \min J(\omega ,\xi )=\frac{1}{2}\omega^{T}\omega +\frac{1}{2}\gamma \sum_{i=1}^{n}\xi_{i}^{2}\\ \begin{array}{cc} s.t. & \begin{array}{c} y(i)[\omega^{T}\varphi (x(i))+b]=1-\xi_{i}\;i=1,2,\cdots ,n \end{array} \end{array} \end{array}$$

where *γ* is the regularization parameter. Through the Lagrange function to solve this issue, the LSSVM optimization turns into Equation (7).

(7) $$\left[\begin{array}{cc} 0 & y^{T}\\ y & \Omega +\gamma^{-1}I \end{array}\right]\left[\begin{array}{c} b\\ a \end{array}\right]=\left[\begin{array}{c} 0\\ Y \end{array}\right]$$

where *I* is the identity matrix, Ω*_ij_*= *K*(*x_i_*, *x_j_*) is the kernel matrix, the Lagrange coefficient *a* = [*a*_1_,*a**_2_***,…*a_n_*]. By solving the linear equation, it can be concluded that:

(8) $$\left\{ \begin{array}{l} \hat{a}=(\Omega +\gamma^{-1}I){}^{-1}(Y-by)\\ \hat{b}=(\Omega +\gamma^{-1}I){}^{-1}Y/\left(\Omega +\gamma^{-1}I\right){}^{-1}y \end{array}\right.$$

where *Y* = *by*+(Ω+*γ*^−1^*I*)^−1^*a*, so the regression model of LSSVM is:

(9) $$y=\sum_{i=1}^{n}{\hat{a}}_{i}K(x,x(i))+\hat{b}$$

The radial basis function (RBF) is chosen as the kernel in this paper, and its expression is *k*(*x*,*x_i_*) = exp(−‖*x*−*x_i_*‖^2^/*σ*^2^). Therefore, *γ* and *σ* are still super parameters to be determined.

### 4.2. The LSSVM Parameter Optimization based on CEDA

The parameter selection of LSSVM will determine its learning performance and generalization ability. At present, the most commonly used parameter selection algorithms are GA and PSO [17,18], but the selection, crossover and mutation of the former are too complex, and the latter is prone to be premature, and the convergence speed of two algorithms is still not ideal.

Therefore, the estimation of distributed algorithm (EDA) model based on chaotic mutation optimization is proposed as a parameter optimization algorithm for LSSVM. EDA is a new evolutionary model that uses learning and sampling of the probability model to replace the selection, crossover and mutation in genetic algorithm (GA). It has excellent global searching ability and convergence speed because of the distribution in solution space described by the probability model, and the population generation from a macro perspective by statistical learning, but that also brings the poor local optimization problem [19]. Therefore, the chaotic mutation is imported in EDA to reconstruct the solution space by using its randomness and ergodicity ability, which guarantees the local optimization and the convergence speed.

After initializing the population and solution space, the evolution can be in progress according to the fitness and mutation probability of individuals in the population. Adopting the linear fitness allocation, which is expressed as follows:

(10) $$fit_{i}=\max sp-sp+\beta (sp-\min sp)\frac{ranking_{i}}{NP}$$

where *sp* is the subpop, max*sp* and min*sp* is the range of *sp*, *β* is the *sp* coefficient, *ranking* is the index of fitness rank for the current individual, *NP* is particles number in the population. It will be mutated and evolved when the new population is becoming monotonous. The individual *x_ijG_* after chaotic mutation can be expressed as:

(11) $$x_{ijG}^{\prime }=x_{ijG}+r_{ij}\alpha_{ij}$$

where *i* is the ordinal of the population, changed from 1 to *NP*, *j* is the dimension of the population, *α_ij_* is the chaotic time series generated by one-dimensional Logistic map, expressed as *α_t_*_+1_ = *λα_t_*(1−*α_t_*), *λ* is the control coefficient, *r_ij_* is the radius of variation, shown as:

(12) $$r_{ij}=C_{j}\frac{1-fit_{i}}{\max fit}$$

where the *C_j_* is the mutation step, which has a wide influence for the algorithm. A larger step is used to quickly retrieve in the solution space at initial stage, and smaller steps later for a fine search. The adaptive sampling method is applied and expressed as follows:

(13) $$C_{j}=\frac{\left\|\max x_{ijG}-\min x_{ijG}\right\|}{DIV}$$

where the *DIV* is the partition coefficient. Substituting Equations (12) and (13) into Equation (11), the new population after chaotic mutation can be obtained.

In addition to the chaotic mutation, the diversity of population also can be improved by reverse operation. While different from a mutation, a reverse operation does not generate new individuals, and a randomly selected sequence which is controlled by reverse probability is rearranged in the original population. Chaotic mutations and reverse operations can ensure the diversity of the population.

The fitness of the population after chaotic mutations and reverse should be reevaluated. The population shoulders the responsibility of producing new generations at this moment, so the fitness evaluation model should also consider the concentration between individuals. Individual concentration is used to describe the number of similar individuals, the individual with high fitness and low individual concentration should be promote the selection probability. Individual concentration can be expressed as:

(14) $$\begin{array}{l} \rho_{i}=\max d_{i}/d_{i}\\ d_{i}=\sum_{l=1}^{NP}\sqrt{{\left(X_{iG}-X_{lG}\right)}^{2}} \end{array}$$

where *d_i_* is the sum of individual concentration. The new fitness evaluation method is shown as follows:

(15) $$fit_{i}^{\prime }=fit_{i}/\rho_{i}$$

**Figure 7:**
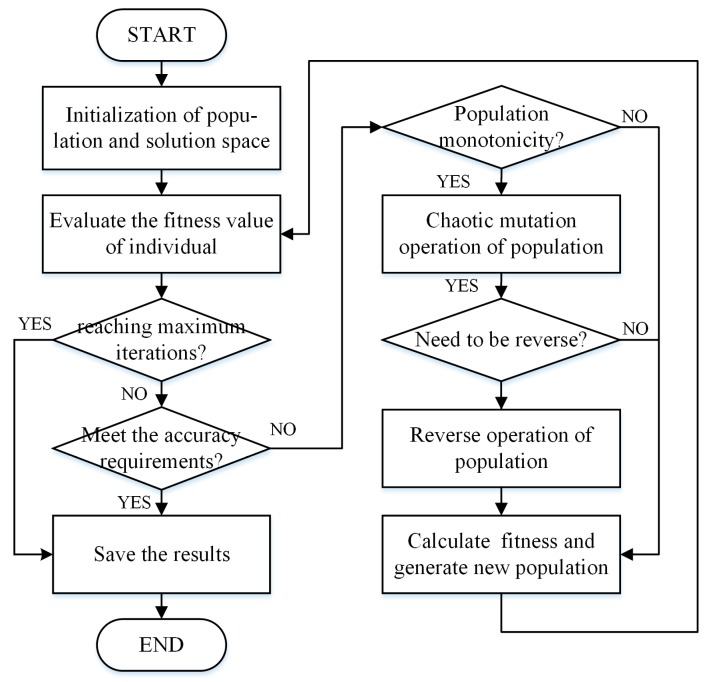
The process of the least squares support vector machine algorithm (LSSVM) parameter optimization by chaos estimation of distributed algorithm (CEDA).

After the fitness evaluation of the population, the new generation is ready. The strategy of generating is to select *np* (*np* < *NP*) better individuals from the population as a judging basis, generate *k* × *NP* (*k* > 1) new individuals through Gauss model, and reselect *NP* individuals as the next generation population. Following this, continue iterating until the fitness value reaches the expectation or the number of iterations reaches the limitation. Therefore, the process of LSSVM parameter optimization by CEDA is shown in Figure 7.

## 5. Numerical Simulation Analysis

### 5.1. Performance Analysis of CEDA

CEDA introduces chaotic mutation to improve the anti-premature ability. It first compares with EDA to verify its effectiveness. Considering the number of parameters to be optimized in LSSVM, ShafferF6 function is chosen as the test function, and its expression is as follows:

(16) $$\begin{array}{c} \min f(x_{1},x_{2})=0.5+\frac{\sin^{2}\sqrt{x_{1}^{2}+x_{2}^{2}}-0.5}{{\left(1+0.001(x_{1}^{2}+x_{2}^{2})\right)}^{2}}\\ \begin{array}{c} s.t.\begin{array}{c} -10\leq x_{i}\leq 10\;\;\begin{array}{c} \left(i=1,2\right) \end{array} \end{array} \end{array} \end{array}$$

**Figure 8:**
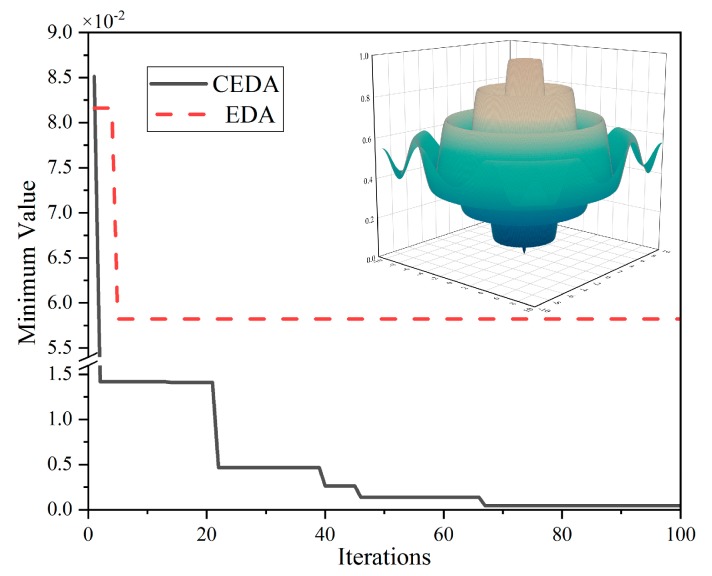
The result of test function extremum finding by CEDA and EDA.

The function has two independent variables, and only one global minimum *f*_min_(0,0) = 0 in its definition domain. In the simulation process, the *NP* of CEDA and EDA is 1000, the maximum number of iterations was 100. The optimal results and the number of iterations are shown in Figure 8.

The attached figure in Figure 8 is the three-dimensional surface graph of the test function. It can be seen that the test function has strong nonlinearity, many local extreme points and only one global minimum point. After the fifth iteration, EDA could not produce better individuals, only evolved ones, and the final fitness was 0.058; CEDA generated six evolutions in 100 iterations, and the final fitness was 4.348 × 10^−4^, which was much better than EDA. 

**Figure 9:**
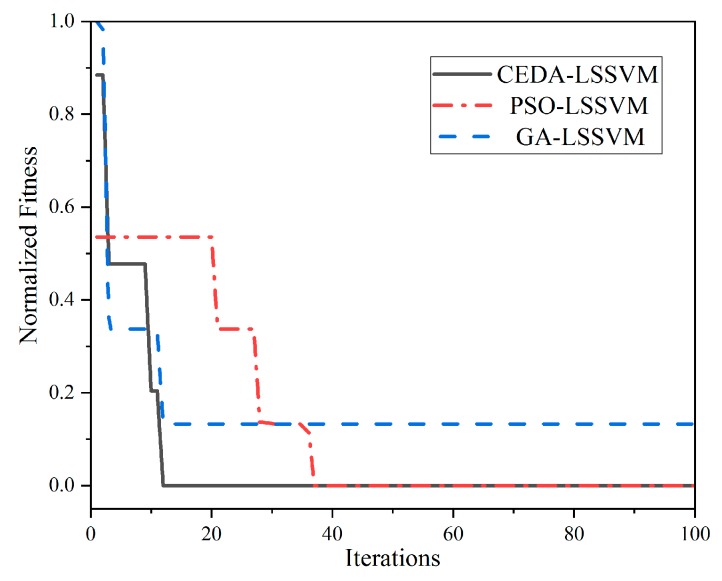
The comparison of the regression effect between CEDA-LSSVM and PSO-LSSVM and GA-LSSVM.

After verifying the advantages of CEDA, the CEDA-LSSVM was compared with GA-LSSVM and PSO-LSSVM. In the simulation process, the *NP* of CEDA was 1000, the population size of PSO and GA was 1000, and the maximum number of iterations was 100. Using the experiment data as regression data, the normalized fitness and the number of iterations curve are shown in Figure 9.

It can be seen from Figure 9 that the convergence speed of GA-LSSVM was better than PSO-LSSVM, but its normalized fitness was lower than PSO-LSSVM. While the convergence speed of CEDA-LSSVM and GA-LSSVM was well matched, but the fitness was slightly better than PSO-LSSVM. Therefore, the gyroscopic effect compensation for SAW torque sensor by CEDA-LSSVM was better than PSO-LSSVM and GA-LSSVM.

### 5.2. Comparison of CEDA-LSSVM with Multivariate Polynomial Fitting (MPF) and Gaussian Processes Regression (GPR)

**Figure 10:**
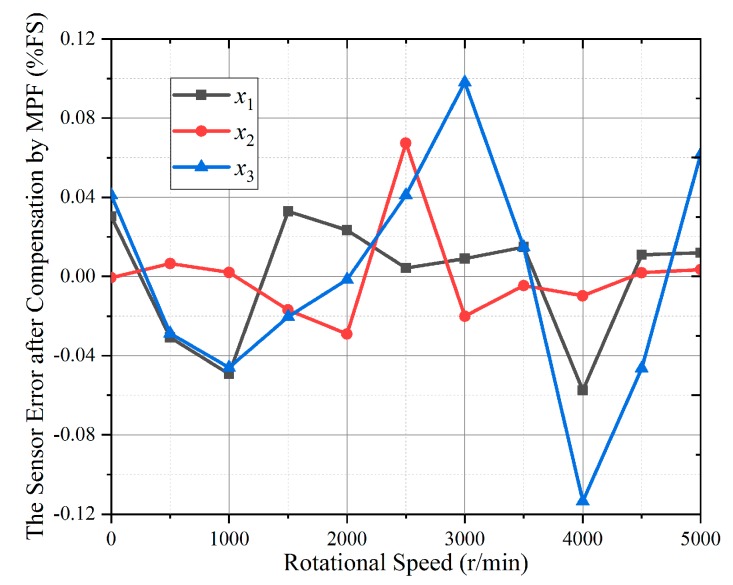
The torque calculation relative errors compensated by multivariate polynomial fitting (MPF).

In Figure 6, the torque calculation errors are obviously non-linear. Therefore, the most commonly used MPF was selected to compensate for the errors. The torque calculation relative errors compensated by MPF are shown in Figure 10.

Considering the complexity and accuracy of the compensation model, the third order for MPF was selected. From Figure 10, it can be seen that the maximum torque calculation relative error in *x*_1_ and *x*_2_ directions are less than 0.06% and 0.08% respectively, but that in *x*_3_ direction is still more than 0.1%. By comparing Figure 10 with Figure 6, it can be seen that MPF had a good compensation effect for the gyroscopic effect error, but that was not ideal in the *x*_3_ direction. The reason is that the data in *x*_3_ direction has the characteristics of irregular with multi peak and valley. To compensate, it should improve the MPF’s order. However, increasing the order will not only increase the computational complexity, but also affect the compensation effect in *x*_1_ and *x*_2_ directions. Therefore, it is necessary to select a more suitable non-linear compensation algorithm to compensate for the gyroscopic effect.

**Figure 11:**
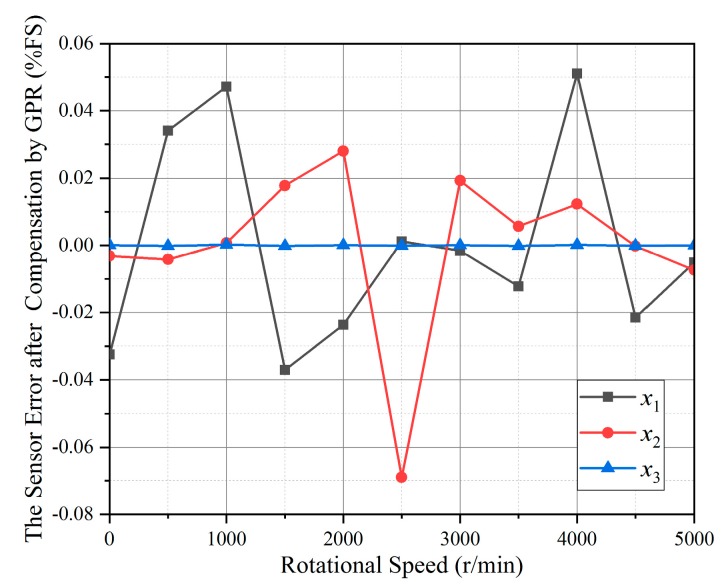
The torque calculation relative error after compensation by Gaussian processes regression (GPR).

Gaussian processes (GPs) are probabilistic kernel machines, which have the characteristics of flexible non-parametric inference, probability interpretation of predictions and easy realization. GPR is moderately simple to implement and use without loss of performance compared with artificial neural networks (ANN) or SVM. Even with a small number of samples, the ability of prediction is still superior [20]. The torque calculation relative error after compensation by GPR is shown in Figure 11.

**Figure 12:**
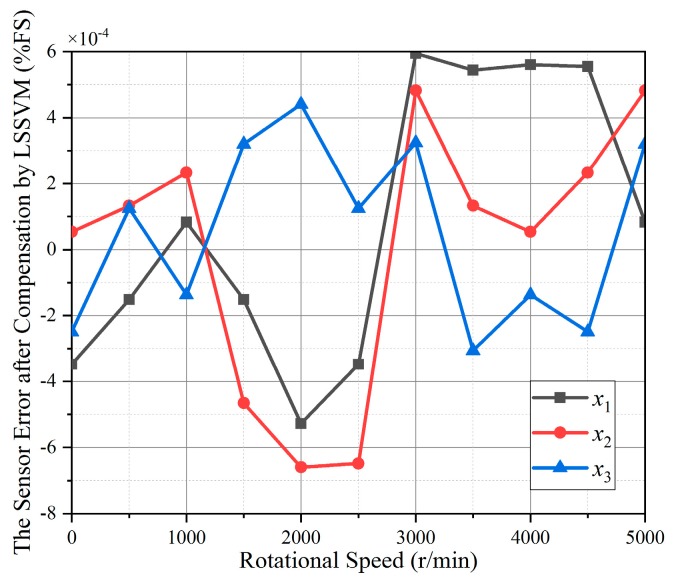
The torque calculation relative error compensated by CEDA-LSSVM.

The squared exponential is selected as the kernel of GPR for prediction and regression. From Figure 11, the maximum torque calculation relative errors in *x*_1_ and *x*_2_ directions were within 0.06% and 0.08% respectively, and the order of magnitude in *x*_3_ direction was 10^−4^. Compared with MPF, the compensation effects in *x*_1_ and *x*_2_ directions are comparable, and significantly improved in *x*_3_ direction. Therefore, the compensation of gyroscopic effect by GPR has a good achievement. Nevertheless, in order to make further improvements on the measurement accuracy of SAW torque sensor, the LSSVM based on CEDA parameter optimization was proposed and used, and the torque calculation relative errors compensated by CEDA-LSSVM are shown in Figure 12.

From the Figure 12, it can be concluded that the magnitude of the torque calculation relative errors in *x*_1_, *x*_2_, and *x*_3_ directions are 10^−4^. The comparisons of maximum torque calculation relative errors after compensation by three algorithms are shown in Table 1. The compensation results of CEDA-LSSVM in *x*_1_ and *x*_2_ directions are much better than MPF and GPR. CEDA-LSSVM in *x*_3_ direction was slightly inferior to GPR, but much better than MPF, and the error magnitude was reduced to 10^−4^. Therefore, the LSSVM based on CEDA parameter optimization can be used to compensate for the influence of gyroscopic effects on the SAW torque sensor.

## 6. Conclusions

In this paper, the SAW torque sensor under high rotational speed environment was studied in depth, the influence of rotation on the SAW torque sensor was analyzed, and the theoretical correctness was verified by experiment. The gyroscopic effect compensation model of the SAW torque sensor was established by LSSVM based on CEDA parametric optimization, and the superiority was verified by comparing with a variety of other algorithms. The experimental results show that the magnitude of the torque calculation relative errors after compensation were 10^−4^, which verified the validity of the proposed compensation method for the gyroscopic effect of the SAW torque sensor, and improved the accuracy and environmental applicability of the SAW torque sensor.

## Figures and Tables

1- Loop Amplifier. 2- Phase Compensator. 3- Low Noise Amplifier. 4- RF Filter. 5- Frequency Mixer. 6- Low-Pass Filter. 7- Clipping Amplifier. 8- Phase-Locked Loop.

**Table 1:** The comparisons of the best compensation result by MPF, GPR and CEDA-LSSVM.

Rotational Axis Direction	Best Compensation Result (%FS)		
	MPF	GPR	CEDA-LSSVM
*x*_1_	0.05755	0.05106	**5.94693 × 10^−4^**
*x*_2_	0.06729	0.06901	**4.40580 × 10^−4^**
*x*_3_	0.11352	**2.20221 × 10^−4^**	6.59945 × 10^−4^
